# Aluminum Spent Foundry Sand as an Emergent Raw Material in the Production of a Sustainable Aluminosilicate Refractory Castable

**DOI:** 10.3390/ma18245500

**Published:** 2025-12-07

**Authors:** Jesús Fernando López-Perales, Leonel Díaz-Tato, Yadira González-Carranza, José Eulalio Contreras de León, Cristian Gómez-Rodríguez, Daniel Fernández-González, Edén Amaral Rodríguez-Castellanos

**Affiliations:** 1 Facultad de Ingeniería Mecánica y Eléctrica (FIME), Universidad Autónoma de Nuevo León (UANL), San Nicolás de los Garza 66450, Mexico; jlopezp@uanl.edu.mx (J.F.L.-P.); leonel.diazat@uanl.edu.mx (L.D.-T.); yadira.gonzalezcn@uanl.edu.mx (Y.G.-C.); jose.contrerasle@uanl.edu.mx (J.E.C.d.L.); 2 Departamento de Mecánica, Facultad de Ingeniería, Universidad Veracruzana Campus Coatzacoalcos (UV), Coatzacoalcos 96535, Mexico; crisgomez@uv.mx; 3 Centro de Investigación en Nanomateriales y Nanotecnología (CINN), Consejo Superior de Investigaciones Científicas (CSIC), Universidad de Oviedo (UO), Principado de Asturias (PA), Avda. de la Vega, 4–6, 33940 San Martín del Rey Aurelio, Asturias, Spain; d.fernandez@cinn.es

**Keywords:** aluminum spent foundry sand, quartz, solid waste, aluminosilicate refractory castable, sustainability

## Abstract

Metal casting industries generate substantial quantities of spent foundry sand (SFS), a silica- and alumina-rich by-product that remains underutilized, with recycling rates below 30%. This study explores the incorporation of aluminum SFS as a secondary raw material in aluminosilicate refractory castables to promote sustainable waste valorization and circular economy practices. Refractory mixtures were prepared with bauxite, kyanite, calcium aluminate cement, microsilica, and flint clay, where fine flint clay was partially replaced by aluminum SFS at 0, 5, 10, and 15 wt.%. Samples were dried at 120 °C and sintered at 850, 1050, and 1400 °C for 4 h. Bulk density, apparent porosity, cold crushing strength, and modulus of rupture were measured, while phase and microstructural evolution were examined by XRD and SEM. The 5 wt.% SFS-containing castable exhibited comparable strength and density to the reference formulation, attributed to the formation of secondary mullite and anorthite that improved matrix cohesion. Higher SFS contents (10–15 wt.%) increased porosity and reduced strength due to excess SiO_2_ and silica polymorphism. These results demonstrate the technical feasibility of using aluminum SFS in refractory castables, contributing to resource conservation, waste reduction, and the development of environmentally sustainable refractory materials for high-temperature applications.

## 1. Introduction

Aluminum is regarded as a crucial engineering material because its density is only about one-third of that of iron and steel. It also combines high specific strength with good corrosion resistance, formability, and recyclability, which reinforces its importance in many industrial applications [1,2,3]. These characteristics make aluminum indispensable for producing lightweight and complex components that enhance structural efficiency and energy performance across various industries. Consequently, it plays a vital role in construction, transportation, energy, and packaging sectors, where its use contributes to improved durability and reduced environmental impact [2,3]. Given global energy and sustainability challenges, the growing demand for lightweight and energy-efficient systems has intensified the use of aluminum-based materials in vehicles and infrastructure to improve fuel economy, reduce greenhouse gas emissions, and promote sustainable manufacturing [4,5].

Global aluminum production has increased by nearly 139% over the past decade, reaching record levels in 2023 [1]. This growth underscores the strategic importance of the aluminum sector but also highlights its environmental challenges. Aluminum smelting and casting are energy-intensive processes that consume vast amounts of electricity and generate significant greenhouse gas emissions, atmospheric pollutants, and solid waste [2]. Among these wastes, large volumes of spent foundry sand are produced by foundries that employ silica-based molds for casting molten aluminum [6]. The disposal of aluminum spent foundry sand (ASFS) poses both technical and environmental challenges, stimulating interest in its sustainable valorization under circular-economy principles.

Foundries worldwide consume hundreds of millions of tons of high-purity silica sand annually as a primary raw material for mold and core fabrication [5]. Depending on the binder system, molding sands are classified as green or chemically bonded. Green sand molds typically consist of 85–95 wt.% silica sand, 4–10 wt.% bentonite clay, 2–10 wt.% carbonaceous additives, and water to provide plasticity and collapsibility [7,8]. Chemically bonded sands typically contain 1–3 wt.% of organic or inorganic binders such as phenolic–urethane, furan, or phenolic–formaldehyde resins, which provide good dimensional accuracy and mechanical strength at high temperatures [7,8]. These binder systems support the production of complex shapes and fast molding operations; however, the thermal degradation of the resins reduces the potential for sand reclamation, and the material is often discarded after only a few reuse cycles.

During casting, the sand molds are exposed to high thermal loads, and subsequent mechanical or thermal reclamation alters the grain morphology, particle size distribution, and surface chemistry [9]. These changes reduce permeability and packing density, leading to casting defects and, ultimately, classification as spent foundry sand (SFS) [10,11,12]. Worldwide production of SFS exceeds 100 million tons each year, yet only about 30% is recovered for reuse, mostly in low-value applications such as concrete, cement, and road construction [3,5,9]. The remaining quantities are typically landfilled, posing environmental risks associated with residual binders, heavy metals, and trace contaminants. Therefore, the development of high-value applications for SFS is essential to mitigate ecological and economic pressures.

Current strategies for SFS reuse have focused on cementitious materials and pavement fillers, guided largely by its physical and chemical resemblance to silica [12,13,14,15]. However, the characteristics of SFS vary significantly depending on its source, binder type, additives, thermal exposure, and the metal alloy cast, all of which affect its composition, contamination level, and morphology [12,13,14,15]. Typically, green SFS contains over 80 wt.% SiO_2_ with minor Al_2_O_3_ and Fe_2_O_3_, while chemically bonded sands exhibit even higher SiO_2_ contents due to the resin systems employed. Mineralogically, SFS consists mainly of crystalline quartz, occasionally accompanied by mullite, zircon, or cristobalite, depending on casting conditions [10,16]. Such compositions confer thermal stability and chemical inertness, suggesting that SFS could serve as a promising precursor for ceramic and refractory applications.

Aluminosilicate refractory castables, especially those classified as conventional or medium-cement formulations, provide an appropriate basis for integrating industrial by-products in alignment with circular-economy goals. These materials typically contain refractory aggregates (e.g., bauxite, chamotte, or kaolinitic clays), calcium aluminate cement binders, and additives to control rheology and sintering [17]. Upon heat treatment, they develop stable crystalline phases such as mullite (3Al_2_O_3_·2SiO_2_), corundum (α-Al_2_O_3_), and silica polymorphs (quartz, cristobalite). Among these, mullite is particularly desirable due to its low thermal expansion, high creep resistance, and superior corrosion and thermal-shock resistance [18]. The interlocking formation of mullite crystals reduces porosity, strengthens grain boundaries, and enhances mechanical performance, making these castables suitable for metallurgical, glass, and advanced ceramics applications [19]. Consequently, any by-product rich in SiO_2_ and Al_2_O_3_ has strong potential as a sustainable raw material for refractory systems [20,21,22,23,24,25,26,27,28,29,30,31,32].

Within this scheme, aluminum spent foundry sand (ASFS) emerges as a promising secondary raw material capable of partially substituting traditional aluminosilicate sources. Its reuse can conserve non-renewable resources, reduce the extraction of virgin clays and silica, and minimize the environmental burden associated with waste disposal and transportation [7]. Despite its favorable chemistry and mineralogy, few studies have examined its incorporation into dense refractory castables, leaving a clear research gap concerning its influence on phase evolution, microstructural development, and high-temperature mechanical behavior.

Among the limited research available, Xiang et al. [33] utilized waste foundry sand containing 46.2 wt.% mullite, 28.1 wt.% cristobalite, and 10.9 wt.% zircon to fabricate refractory bodies using 10 wt.% polyvinyl alcohol binder. The samples exhibited good stability up to 1450 °C; however, higher firing temperatures induced the tetragonal-to-monoclinic transformation of ZrO_2_ and partial melting of cristobalite, which reduced mechanical strength. Similarly, Bathli et al. [19] investigated chamotte-based refractory concrete incorporating 5–20 wt.% of waste sand from steel casting. Additions up to 10 wt.% improved densification by reducing porosity, but the study did not evaluate mechanical performance, leaving the structure–property relationships unresolved.

This study examines the use of aluminum spent foundry sand (SFS) from automotive casting operations as a sustainable partial replacement for flint clay in aluminosilicate refractory castables. The central hypothesis is that aluminum SFS supplies additional silica during sintering, which favors the formation of a SiO_2_-rich liquid phase. This phase is expected to enhance mullite crystallization and strengthen interparticle bonding. If this mechanism is confirmed, the castables should develop a more uniform microstructure, fewer defects, and mechanical and thermal properties comparable to those of conventional materials.

To test this hypothesis, the investigation evaluates the effects of aluminum SFS on crystallography, microstructure, and physicomechanical performance across different firing temperatures. The specific objectives are as follows:

(i) to characterize microstructural development using SEM/EDS and relate secondary mullite morphology and phase distribution to mechanical behavior;
(ii) to determine the influence of SFS on bulk density, porosity, cold crushing strength, and modulus of rupture in order to identify the most suitable substitution level;
(iii) to assess the compatibility of aluminum SFS with standard refractory formulations, with particular attention to sintering and densification; and
(iv) to establish links between microstructural features and property evolution to clarify the mechanisms that govern mechanical integrity and thermal stability.

In addition to its technical relevance, this study highlights the environmental benefit of using an aluminum industry by-product as a functional raw material for refractory production. Partial replacement of flint clay with aluminum SFS reduces the use of natural resources and supports a circular approach to refractory production. The outcomes indicate the potential of this material to meet the requirements of high-temperature applications, including those found in biomass combustion systems.

## 2. Materials and Methods

### 2.1. Raw Materials

A conventional aluminosilicate refractory castable was prepared using industrial raw materials supplied by AP Green (Salinas Victoria, Nuevo León, Mexico). The formulation included calcined flint clay, calcined Guayanese bauxite, kyanite containing 48% Al_2_O_3_, calcium aluminate cement (Secar 80), and microsilica.

To enhance the environmental profile of the mixture, finely ground aluminum spent foundry sand (<75 µm, obtained through mechanical sieving) was incorporated as a secondary raw material. The material was supplied by the Nemak foundry in Garcia, Nuevo Leon, Mexico, which employs silica-based molds for aluminum casting. This waste was selected because it offers a practical route to valorize ceramic residues while lowering the use of primary refractory resources.

The chemical compositions of the standard raw materials and the aluminum spent foundry sand were determined by X-ray fluorescence using a Panalytical Magix PW2424 spectrometer (Philips, Malvern Panalytical Ltd., Malvern, UK). Before analysis, each powder was dried, homogenized, and sieved to below 75 µm. The samples were prepared using a standard pellet-press procedure. The powders were mixed with a small amount of analytical-grade binder, placed into a steel die, and pressed at high pressure to produce flat, compact pellets suitable for XRF measurement. This preparation method ensured consistent sample density and minimized surface irregularities. The oxide contents are reported in the Results section.

Crystalline phases were identified using a Bruker D8 Advance diffractometer (Bruker AXS GmbH, Karlsruhe, Germany) with CuKα radiation (λ = 1.5406 Å, 40 kV, 45 mA) and a PIXcel 1D detector (Malvern Panalytical, Almelo, The Netherlands). Measurements were performed in continuous mode from 10° to 90° (2θ) with a step size of 0.05° and a dwell time of 1.5 s per step.

The phase analysis, presented in the Results section, provided detailed information on the mineralogical composition and amorphous fraction of the materials. This characterization was essential to evaluate their reactivity and their contribution to the microstructural development of the castables.

#### Method of Recycling Aluminum SFS

Prior to its use, the as-received aluminum SFS was processed to ensure particle uniformity and remove residual contaminants. The material was subjected to mechanical grinding using a Vibro-Energy grinding mill (Model DM-1, SWECO Manufacturing, Florence, KY, USA). The instrument used had a 0.5 HP motor and operated at ~1200 rpm loaded with 30 kg of alumina balls (diameter range: 10–60 mm) and operated for 4 h. The milled powder was subsequently sieved through a 200-mesh (75 μm) stainless-steel screen to obtain fine particles suitable for castable formulations. This treatment aimed to enhance homogeneity, improve particle surface activity, and promote better interaction with the binder matrix during the hydration and sintering stages.

### 2.2. Refractory Formulation and Specimen Preparation

For this study, a reference (control) refractory formulation previously developed and reported by the authors [24] was used to produce a conventional aluminosilicate castable (CC). As was previously indicated, the composition was designed using commercially available raw materials, including calcined flint clay, calcined Guayanese bauxite, kyanite (48 wt.% Al_2_O_3_), calcium aluminate cement (Secar 80), and microsilica. In addition, three modified formulations were prepared in which aluminum spent foundry sand (SFS) partially replaced the fine fraction of flint clay at substitution levels of 5, 10, and 15 wt.%. These compositions were designated as 5FSC, 10FSC, and 15FSC, respectively, according to the percentage of SFS incorporated. The substitution levels of 5, 10, and 15 wt.% aluminum spent foundry sand (SFS) were selected based on preliminary optimization trials and the compositional characteristics of the waste material. Previous research on refractory castables that include silica-rich industrial by-products, such as waste molding sands and glass powder, has shown that small additions can promote sintering and improve microstructural stability by strengthening the interaction between silica and alumina within the matrix. Considering these findings, the present work extended the replacement range up to 15 wt.% SFS to systematically examine how increasing the content of this secondary raw material influences densification behavior, phase formation, and mechanical performance at elevated temperatures. Initial experimental batches revealed that additions beyond 15 wt.% noticeably increased water demand and reduced workability, which in turn affected mold filling and green strength. Therefore, the 5–15 wt.% range was established as an optimal interval to evaluate the threshold at which SFS promotes microstructural development and phase stability without compromising the processing performance of the refractory castables.

The raw materials used for designing the SFS-containing conventional refractory castables, together with their corresponding proportions, are summarized in Table 1. The materials are described as follows:

Flint clay aggregates in coarse (4–6.3 mm), medium (2–4 mm and <2 mm), and fine (milled base, MB) fractions. Bauxite aggregates in medium (2–4 mm and <2 mm) and fine (MB) fractions. Additives: kyanite 48 (used as a reactive aluminosilicate to promote mullite formation) and microsilica (to improve particle packing and matrix densification). Binder: Secar 80 calcium aluminate cement, serving as the hydraulic binding phase to ensure mechanical strength development at intermediate curing stages. Secondary raw material: aluminum spent foundry sand (SFS) with a particle size ≤75 μm, collected from aluminum automotive casting operations at Nemak in Garcia, Nuevo Leon, Mexico.

The preparation of the refractory samples followed a systematic two-stage mixing process to ensure homogeneity and reproducibility. Initially, the dry mixing of the starting powders was carried out using a conventional mortar mixer for 3 min according to the proportions listed in Table 1. Subsequently, wet mixing was performed for an additional 4 min after adding the required amount of water (11–11.5 wt.%) to obtain the proper flow and workability for each castable formulation. Water was added following the ASTM C860 (2000) [34] procedure to achieve proper mixing and workability.

The water-to-cement ratio strongly influences the rheological behavior and mechanical performance of refractory castables, with conventional dense formulations typically requiring 8–15 wt.% water relative to dry solids. In this study, 9%, 11%, and 12% water additions were tested to determine the optimal mixing ratio. The consistency of each batch was assessed using the ASTM C860 “Ball-in-Hand” method, widely adopted in industrial practice to evaluate the plasticity and cohesiveness of fresh castables. The mixture with 9 wt.% water showed poor cohesion and inadequate flow, while the 12 wt.% formulation exhibited excessive fluidity and segregation. The 11–11.5 wt.% water content produced a homogeneous and workable mix, ensuring effective compaction under vibration and uniform mold filling. This proportion was therefore selected as optimal, balancing flowability, green strength, and dimensional stability, and ensuring dense microstructure formation after sintering. Maintaining this ratio minimizes defects such as shrinkage, microcracking, or incomplete compaction, leading to improved mechanical strength, thermal shock resistance, and long-term durability under high-temperature service.

The resulting fresh mixtures were poured into plastic molds (50 × 50 × 50 mm^3^ and 25 × 25 × 150 mm^3^), which had been lightly lubricated to facilitate demolding. Each mold was placed on a vibrating table (Model 55-C016/Hz, CONTROLS S.p.A Manufacturing, Milan, Italy) and subjected to vibration for 3 min to remove entrapped air bubbles and ensure compactness. Following casting, the molds were covered with a transparent polymer film and allowed to cure under laboratory ambient conditions (≈25 °C, 50–60% RH) for 24 h to minimize moisture loss through evaporation.

After curing, the castables were demolded and placed in a Thermo Scientific Heratherm drying oven (Thermo Fisher Scientific, Waltham, MA, USA) at 120 °C for 24 h to complete the dehydration stage. The dried (“green”) samples were then subjected to thermal treatment at three different firing temperatures: 850 °C, 1050 °C, and 1400 °C, using a Thermo Scientific Lindberg Blue M/1700 electric furnace (Thermo Fisher Scientific, Waltham, MA, USA). Heating was conducted at a constant rate of 170 °C h^−1^, followed by a 4 h soaking period at the target temperature to allow sufficient sintering and phase evolution. Cooling was carried out naturally by convection within the electric furnace in accordance with ASTM C865 (2002) [35].

The initial treatment at 110 to 120 °C is not a sintering step. Its purpose is to remove free and physically adsorbed water in a controlled manner so that the specimens remain dimensionally stable before higher temperature heating. This drying level is also recommended in ASTM C865 as a standard condition for measuring density, porosity, and strength in refractory castables.

The firing temperatures of 850, 1050, and 1400 °C were selected because they represent fundamental thermal intervals that govern phase evolution in aluminosilicate refractory materials. Heating to about 850 °C allows the dehydroxylation of kaolinite and the formation of metakaolinite. At this stage, calcium aluminate hydrates in the cement matrix decompose, early glass formation begins, and the first significant microstructural rearrangement takes place. This makes 850 °C an appropriate point for evaluating the onset of shrinkage, loss of strength, and early changes in porosity.

The 1050 °C level corresponds to the transformation of metakaolinite into spinel-type phases and silica-rich glass. It also marks the beginning of primary mullite formation. This temperature was chosen to monitor how early mullitization affects densification and mechanical behavior and to simulate medium-temperature service conditions typical of biomass energy systems and ceramic processing equipment.

The 1400 °C treatment represents the high-temperature consolidation stage. Secondary mullite and anorthite develop at this level, and the microstructure approaches its maximum degree of densification and bonding. Assessing the material at 1400 °C provides information relevant to severe industrial applications involving slag exposure, alkali attack, and high mechanical loads.

This selection of temperatures enables a consistent and systematic evaluation of how flint clay substitution affects the physical, mechanical, and microstructural development of the castables throughout their full thermal cycle.

The procedures for assessing the workability of fresh castables, as well as the casting, curing, and firing regimes, were performed in accordance with ASTM C600 [36], ASTM C862 [37], and ASTM C865 [35] standards, respectively, ensuring consistency with established practices for conventional aluminosilicate refractory castables.

### 2.3. Characterization Techniques

The physical properties, including bulk density and apparent porosity, were measured for the aluminosilicate refractory castables after drying and at each firing temperature. A minimum of six cubic specimens was tested for every condition. Similarly, cold crushing strength (CCS) and cold modulus of rupture (CMOR) were determined using six cubic and six prismatic samples, respectively. Bulk density and porosity were measured by the boiling water method based on Archimedes’ principle, following ASTM C20 (2000) [38], while mechanical tests were performed using an ELE-International hydraulic universal testing machine (ELE International, Leighton Buzzard, UK), with a maximum capacity of 250 kN. CCS was determined using a loading rate of 31.2 kN/min, while the CMOR was assessed via three-point bending on rectangular prism specimens at a loading rate of 0.774 kN/min, following ASTM C133 (1997) [39]. Results were reported as mean values ± standard deviation, based on six replicates.

The phase composition evolution of both the reference and SFS-containing castables were analyzed by X-ray diffraction (Bruker D8 Advance, Bruker AXS GmbH, Karlsruhe, Germany, CuKα, λ = 1.5406 Å, 40 kV, 45 mA) equipped with a PIXcel 1D detector (Malvern Panalytical, Almelo, The Netherlands). Scans were conducted in continuous mode from 10° to 90° (2θ), with a 0.05° step and 1.5 s dwell time per step. Powdered samples (<45 µm) were obtained by grinding portions of fractured specimens after compression tests. The crystalline phases identified were matched to the Crystallographic Open Database (COD) cards. The phase’s quantification of these samples was performed by an X-ray data refinement under Rietveld conditions, using the MAUD (Materials Analysis Using Diffraction) software, version 2.995.

Microstructural observations were performed using SEM (JSM-6510LV JEOL, Ltd., Tokyo, Japan) equipped with an EDS detector (Apollo X, EDAX Inc., Mahwah, NJ, USA) to assess phase distribution and morphology. Prior to analysis, samples were cold-mounted in epoxy resin and sequentially polished using silicon carbide abrasive papers (grits 80–4000) followed by diamond suspensions (9, 3, and 1 µm). The polished surfaces were coated with a thin layer of gold using a sputter coater (Q150R ES, Quorum Technologies Ltd., located in Lewes, East Sussex, UK) to ensure surface conductivity. The results obtained at 1400 °C were correlated with the phase composition and microstructural development to demonstrate their influence on the refractory’s density and mechanical performance.

## 3. Results

### 3.1. Characterization of Raw Materials and Aluminum SFS

Table 2 shows the chemical composition of the standard raw materials and the aluminum spent foundry sand (SFS) analyzed by X-ray fluorescence spectroscopy.

Table 3 summarizes the crystalline phases identified in the raw materials, including the aluminum spent foundry sand.

**Figure 1:**
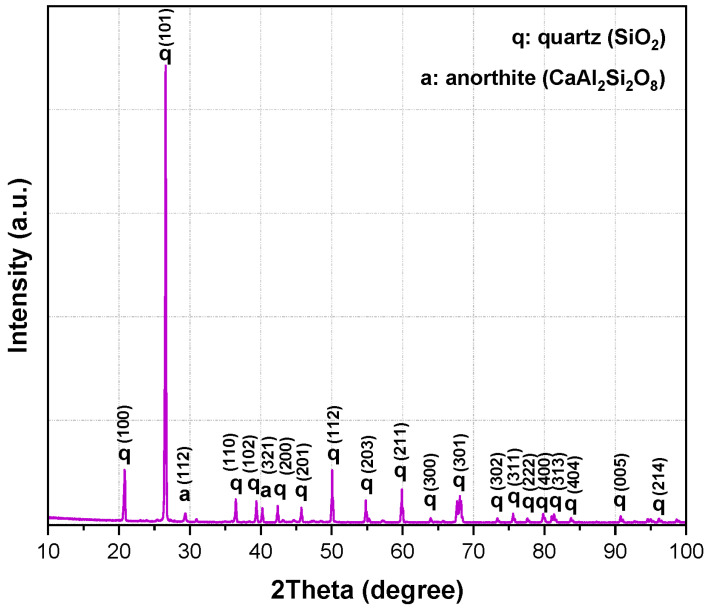
Mineralogical composition of the aluminum spent foundry sand (aluminum SFS).

The combined XRF and XRD results show that the aluminum SFS is mainly composed of SiO_2_, present predominantly as quartz, with smaller amounts of Al_2_O_3_, CaO, and Fe_2_O_3_. This composition reflects the nature of the sand used in aluminum casting. The detected oxides indicate that the material may take part in chemical reactions with the calcium aluminate cement and the aluminosilicate matrix during firing. The main reflections correspond to quartz (SiO_2_, ICDD 01-087-2096) and anorthite (CaAl_2_Si_2_O_8_, ICDD 01-070-0287), which are commonly found in aluminosilicate refractories and are valued for their mechanical stability, thermal resistance, and moderate thermal conductivity (Figure 1).

Calcined flint clay contained mainly mullite (Al_2.272_Si_0.728_O_4.864_, ICDD 01-083-1881) and cristobalite (SiO_2_, ICDD 01-087-2096), along with minor rutile (TiO_2_). In contrast, calcined bauxite, known for its high alumina content, showed corundum (α-Al_2_O_3_, ICDD 00-043-1484), mullite, aluminum titanate (Al_2_TiO_5_, ICDD 01-076-8797), and traces of quartz.

These phases contribute to high refractoriness and chemical durability. Kyanite (Al_2_SiO_5_, ICDD 01-072-1447) presented reflections of its characteristic phase together with quartz and rutile. Since kyanite converts to mullite and cristobalite when heated, its presence supports dimensional stability and bonding at elevated temperatures. The calcium aluminate cement Secar 80 contained calcium monoaluminate (CA, ICDD 01-076-7124), calcium dialuminate (CA_2_, ICDD 01-089-3851), and corundum. These phases explain the hydraulic setting behavior at room temperature and provide early mechanical strength before sintering. Microsilica displayed the broad halo typical of amorphous silica, confirming its role as a reactive additive. Its amorphous nature improves particle packing and contributes to the formation of secondary mullite during firing, which enhances bonding, reduces porosity, and improves the high-temperature performance of the castables.

### 3.2. Physical Characteristics of Refractory Castables

**Figure 2:**
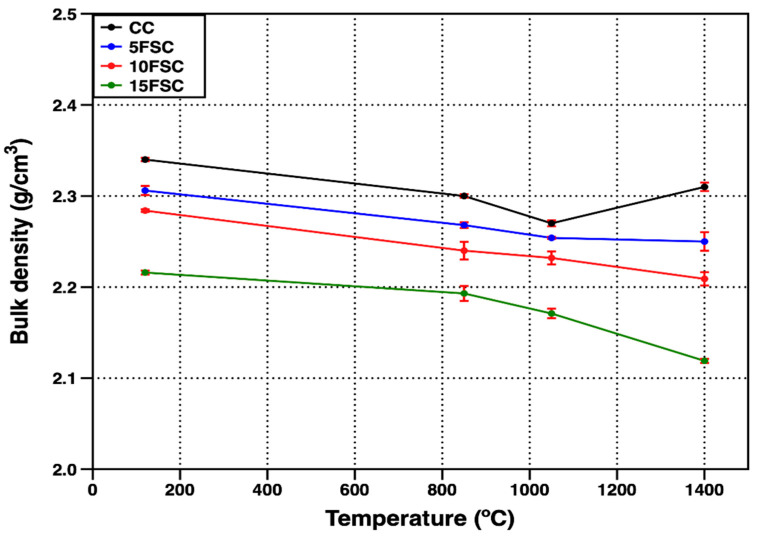
Effect of aluminum spent foundry sand on the bulk density of refractory castables as a function of firing temperature.

Figure 2 presents the bulk density of the reference castable and the SFS-containing aluminosilicate castables as a function of firing temperature. At the drying stage (120 °C), the reference sample exhibited the highest bulk density (2.34 g/cm^3^), while a progressive decrease was observed with increasing substitution of flint clay by aluminum SFS. The 5FSC, 10FSC, and 15FSC formulations displayed densities of 2.30, 2.28, and 2.21 g/cm^3^, respectively. This gradual decrease in bulk density is mainly explained by the partial substitution of flint clay, which usually has a density of 2.0–2.7 g/cm^3^, with aluminum SFS. This byproduct is lighter and has an apparent density in the range of 1.53–1.78 g/cm^3^. Additionally, SFS-containing mixtures required a higher water-to-solid ratio to achieve adequate flowability, which likely increased the amount of residual porosity after drying due to water evaporation, thereby contributing to the observed density reduction [7,40].

As the firing temperature increased, all castables exhibited a further decline in bulk density, particularly at intermediate temperatures. This effect was more pronounced for the reference and 15FSC compositions between 850 °C and 1050 °C compared with 5FSC and 10FSC. Within this range, the physically adsorbed and capillary water is completely removed, while dehydration and phase conversion processes occur in the calcium aluminate binder system. Specifically, the transformation of metastable hexagonal CAH_10_ and C_2_AH_8_ into the stable cubic C_3_AH_6_ phase leads to the release of chemically bound water, generating additional porosity and volume contraction that collectively reduce the apparent density of the ceramic matrix [41].

Moreover, the incorporation of aluminum SFS introduces additional quartz into the refractory composition, which undergoes a polymorphic transition from α-quartz to β-quartz near 573 °C. This transformation is accompanied by a sudden volume expansion that can induce microcracking and localized pore formation within the matrix, further promoting the decline in density with temperature [42]. At higher SFS levels, the combined effects of increased quartz content, elevated porosity from water loss, and incomplete sintering consolidation result in a more open microstructure, consistent with the observed density trend.

At 1400 °C, the reference castable exhibited a slight increase in bulk density, reaching 2.31 g/cm^3^, suggesting the onset of sintering and the formation of a stable ceramic bond that effectively reduced residual porosity by tightening the particle packing within the matrix. This densification process is characteristic of conventional aluminosilicate castables, where vitrification and solid-state diffusion promote bonding among aggregates and fine fractions. In contrast, the 5FSC, 10FSC, and 15FSC castables showed a decrease in bulk density at the same firing temperature, recording values of 2.25, 2.21, and 2.11 g/cm^3^, respectively. This opposite trend indicates that, in SFS-containing compositions, the beneficial densification associated with sintering was counteracted by microstructural instability induced by the polymorphic transitions of silica (α- to β-quartz and β-quartz to cristobalite). These transitions involve abrupt volume changes that can generate internal stresses, cracking, and localized pore formation. The effect becomes more pronounced in the 15FSC formulation, which contains the highest silica content derived from the spent foundry sand, thus intensifying the expansion–contraction cycles during heating and cooling [42]. Consequently, the presence of a higher amount of SiO_2_-rich particles hindered the overall consolidation of the ceramic matrix and limited the densification typically expected at elevated firing temperatures.

**Figure 3:**
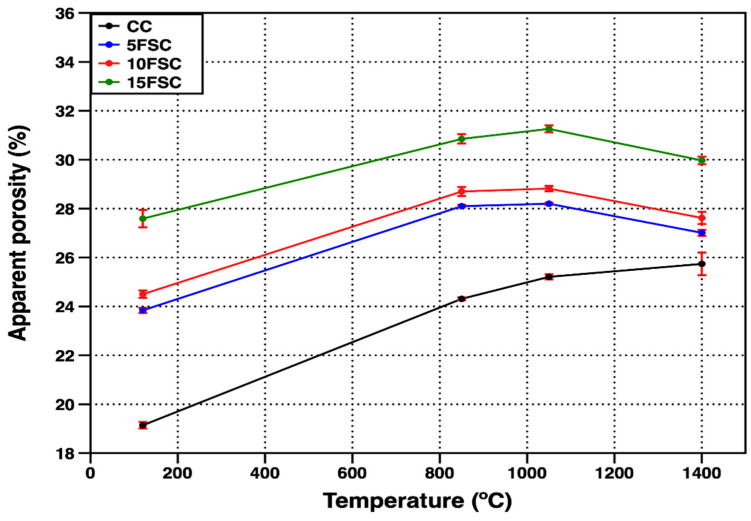
Effect of aluminum spent foundry sand on the apparent porosity of refractory castables as a function of firing temperature.

Figure 3 shows the evolution of apparent porosity for both the reference and SFS-containing refractory castables as a function of temperature. The observed porosity trends align closely with the bulk density results. At the drying temperature (120 °C), the reference castable, which showed the highest bulk density (2.34 g/cm^3^), also exhibited the lowest open porosity (19.14%). Conversely, all SFS-containing formulations displayed higher open porosity values, primarily due to the incorporation of a lower-density secondary raw material and the higher water content required to ensure workable consistency during casting [43,44].

According to Alonso-Santurde [40], the inclusion of spent foundry sand in clay-based or aluminosilicate matrices decreases plasticity, necessitating greater water addition and leading to increased porosity after drying and firing. This effect is particularly evident in the 15FSC sample, which required the highest water addition and consequently presented the highest open porosity (27.59%). Increasing the firing temperature to 850 °C and 1050 °C resulted in a progressive rise in apparent porosity across all formulations, with a more pronounced increase in the reference and 15FSC castables. This behavior can be associated with the dehydration of calcium aluminate hydrates and the structural rearrangement of silica phases, both of which contribute to pore coalescence and volumetric expansion within the microstructure.

The combined density and porosity results suggest that the introduction of SFS modifies the sintering dynamics of aluminosilicate refractory castables. While moderate additions (≤10 wt.%) maintain acceptable densification and pore control, higher levels (15 wt.%) promote excessive silica-driven phase transformations and structural discontinuities that compromise the compactness and homogeneity of the fired material.

During the initial heating stages, conventional aluminosilicate castables primarily experience the removal of physically adsorbed moisture and the dehydration of calcium aluminate hydrates, leading to the formation of additional pores and voids within the microstructure. These processes are responsible for the transient increase in porosity commonly observed at intermediate temperatures. In contrast, for the SFS-containing formulations, the microstructural evolution is further influenced by the polymorphic transformation of silica during the cooling stage. Specially, the transformation of β-quartz to α-quartz is accompanied by a sudden reduction in volume, which generates internal stresses and promotes the formation of microcracks within the matrix. This effect becomes more pronounced when flint clay is partially replaced by aluminum spent foundry sand, since this material adds a larger amount of crystalline SiO_2_, mainly in the form of quartz. As a result, the structural instability linked to these phase transformations increases significantly [45,46,47,48,49].

At 1400 °C, the open porosity of the reference castable exhibited a distinct trend compared to the SFS-containing formulations. Although the reference material showed a slight increase in bulk density at this temperature, its apparent porosity also increased slightly, reaching 25.74%. This apparent contradiction suggests that the ceramic bonding phase was only partially developed, resulting in limited cohesion between the matrix and the coarse aggregates. Consequently, some microvoids persisted despite partial densification, reflecting an incomplete sintering process and insufficient liquid-phase formation to fully consolidate the refractory body.

In contrast, the SFS-containing castables exhibited a reduction in open porosity at the highest firing temperature, with 5FSC and 10FSC samples achieving porosity levels similar to that of the reference formulation (27.01% and 27.62%, respectively). This improvement can be attributed to the generation of a low-viscosity liquid phase rich in SiO_2_, derived from the SFS particles, which also contain minor impurities such as Al_2_O_3_, Fe_2_O_3_, and CaO [14,50]. Upon heating, these oxides react to form transient liquid films that enhance particle rearrangement and diffusion at grain boundaries, promoting the development of a stronger ceramic bond. As sintering progresses, aluminosilicate phases, especially mullite, begin to nucleate and develop within the matrix. These phases form interlocking acicular crystals that extend across the matrix and aggregate interfaces. The growth and interconnection of the mullite needles reduce the number of continuous pores, strengthen the microstructural network, and lower the open porosity of the fired specimens.

The observed behavior aligns with the findings of Mohamed et al. [13], who reported that incorporating SiO_2_-rich by-products such as silica fume into ceramic matrices reduced both bulk density and porosity at high temperatures due to enhanced liquid-phase sintering. In the present work, a similar mechanism appears to govern the densification of SFS-containing refractory castables. The balance between silica enrichment and liquid-phase development plays a crucial role in determining the extent of densification and pore closure. Moderate additions of SFS (≤10 wt.%) favor this effect by promoting controlled liquid-phase formation without excessive expansion or structural weakening, while higher additions tend to induce detrimental volumetric instability during cooling.

### 3.3. Crystalline Phase Evolution of Refractory Castables

**Figure 4:**
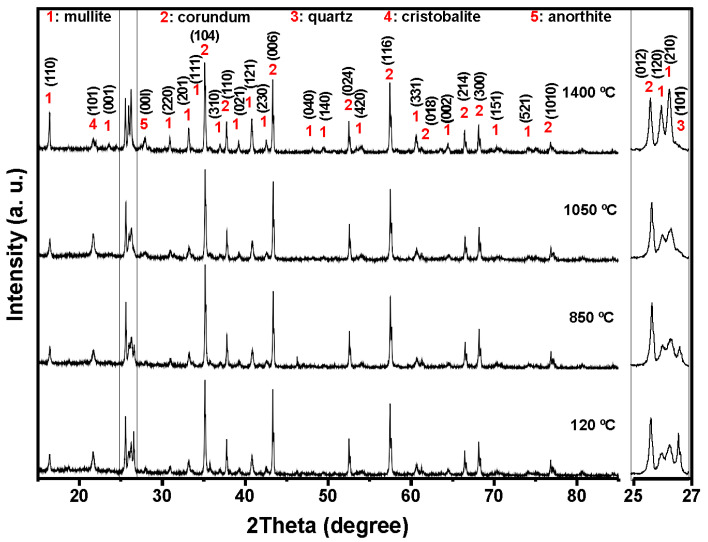
Evolution of crystalline phases as a function of firing temperature for the reference refractory castable.

**Figure 5:**
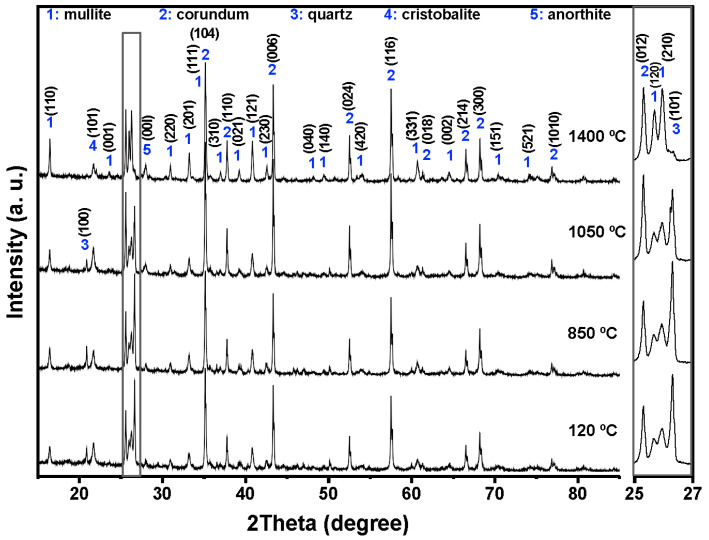
Evolution of crystalline phases as a function of firing temperature for the 5FSC refractory castable.

**Figure 6:**
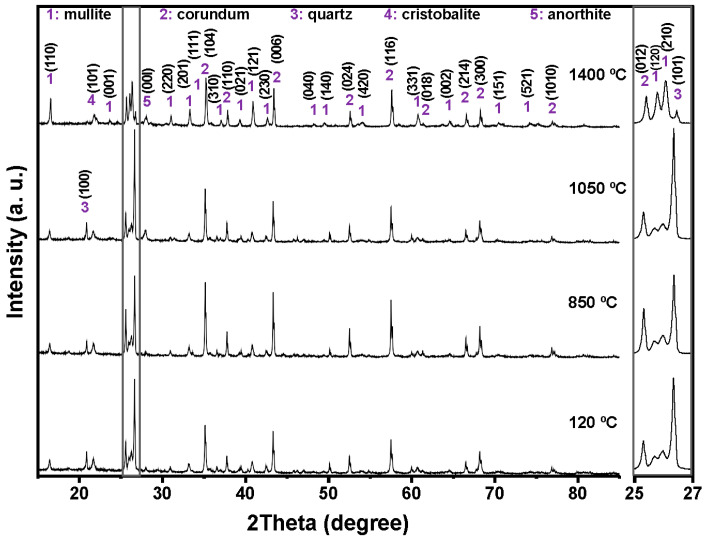
Evolution of crystalline phases as a function of firing temperature for the 10FSC refractory castable.

**Figure 7:**
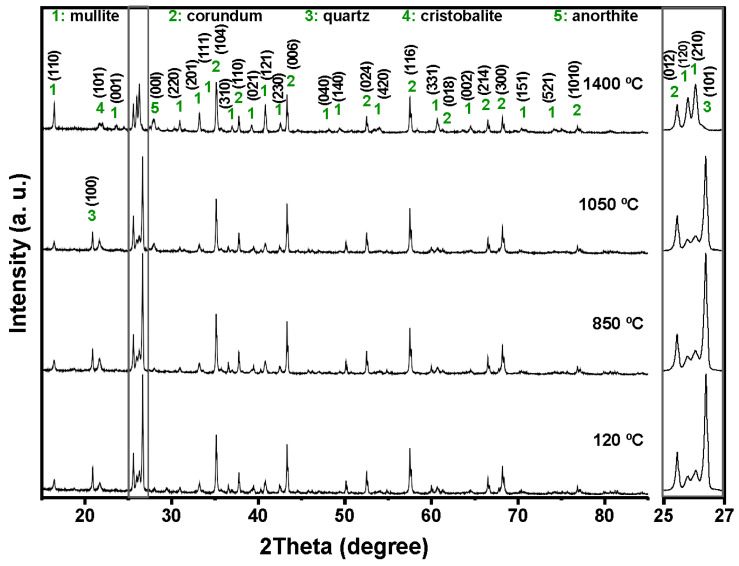
Evolution of crystalline phases as a function of firing temperature for the 15FSC refractory castable.

Figure 4, Figure 5, Figure 6 and Figure 7 shows the crystalline phases identified and the evolution of crystalline phases in the reference castable and the SFS-containing formulations examined at 120, 850, 1050, and 1400 °C. The Rietveld refinement results (Table 4) were used to support the qualitative observations obtained from the diffractograms.

At 120 °C, all formulations displayed the initial phase assemblage derived from the raw materials, i.e., mullite (Al_2.272_Si_0.728_O_4.864_, ICDD 01-083-1881), corundum (α-Al_2_O_3_, ICDD 01-088-0826), cristobalite (SiO_2_, ICDD 01-082-0512), and quartz (SiO_2_, ICDD 01-087-2096) [51,52]. The reference castable contained mainly corundum (60.26 wt.%) and mullite (30.48 wt.%), together with small amounts of cristobalite and quartz.

Incorporation of SFS increased the amount of crystalline SiO_2_ in the system, as observed in the 2θ region between 20 and 23° and in the magnified range from 25 to 27°. The quartz content ranges from 4.81 wt.% in the reference castable to 12.46 wt.% in 5FSC and 21.06 wt.% in 10FSC. A smaller increase was observed in 15FSC (19.93 wt.%). These results confirm that SFS introduced additional quartz residues from the foundry sand. Corundum and mullite contents decreased proportionally due to the higher silica fraction supplied by SFS.

Heating to 850 °C produced distinct changes in the reference formulation. Quartz and cristobalite decreased (2.53 wt.% and 1.56 wt.%), while corundum increased to 63.94 wt.%. This behavior indicates partial dissolution of silica phases and the progressive development of alumina-rich phases within the refractory matrix.

SFS-containing castables behaved differently due to their higher SiO_2_ content. Quartz remained significantly higher in 5FSC, 10FSC, and 15FSC (14.45, 15.13, and 22.41 wt.%, respectively). Cristobalite showed only minor changes. Mullite contents were slightly higher in SFS-containing mixtures than in the reference (31.95 wt.% in the reference vs. 34.93 wt.% in 5FSC). The combination of elevated quartz and modest cristobalite development suggests that silica from SFS persisted in crystalline form at this temperature and was only partially involved in mullite formation. A higher SiO_2_ content favored the α→β transformation of quartz and its subsequent recrystallization into cristobalite. These polymorphic conversions, characteristic of silica-rich systems within this temperature range, involve significant volume changes that can induce microcracking and porosity in the microstructure, adversely affecting density and mechanical integrity [45,53,54].

At 1050 °C, the reference castable showed clear progress toward high-temperature phase formation. Quartz was almost fully consumed (1.11 wt.%), cristobalite increased to 2.40 wt.%, and anorthite (CaAl_2_Si_2_O_8_, ICDD 01-070-0287) appeared at 7.12 wt.%. These transformations indicate active reactions between SiO_2_, Al_2_O_3_, and CaO (originating from the calcium aluminate cement), which favor the growth of mullite and calcium aluminosilicate phases [42,55]. This transformation reflects the onset of solid-state diffusion and partial liquid-phase sintering within the aluminosilicate matrix.

The SFS-containing castables exhibited more complex behavior due to their larger silica supply. In 5FSC, cristobalite increased to 5.20 wt.%, while quartz remained comparatively high (8.37 wt.%). Anorthite was also present at 2.70 wt.%. Anorthite reflections confirms that a SiO_2_-rich liquid phase originating from the SFS reacted with fine Al_2_O_3_ and CaO particles to form calcium aluminosilicate compounds within the matrix. The presence of this phase suggests enhanced reactivity due to SFS addition, as the fine silica particles promote local diffusion processes and facilitate the development of a coherent ceramic bond. In 10FSC, quartz reached its maximum value for this composition (20.36 wt.%), while anorthite increased to 7.80 wt.%. These results show that part of the alumina-rich phase reacted with silica and CaO to form calcium aluminosilicates. In 15FSC, quartz remained high (18.44 wt.%), cristobalite increased to 6.92 wt.%, and anorthite appeared at 7.78 wt.%. However, mullite decreased to 18.11 wt.%. The relatively low mullite content suggests that the excess silica promoted the formation of a viscous SiO_2_-rich liquid that limited solid-state diffusion and restricted anorthite crystallization.

The formation of anorthite (CaAl_2_Si_2_O_8_) in these systems can proceed through two principal mechanisms: (i) transformation of gehlenite (Ca_2_Al_2_SiO_7_) into anorthite through Si^4+^ ion diffusion at high temperatures, or (ii) direct crystallization of anorthite from a CaO-bearing glassy phase generated during thermal treatment. In the present work, the absence of gehlenite peaks in the diffractograms at intermediate firing temperatures supports the second mechanism as the dominant route. Therefore, anorthite in the SFS-containing castables most likely originated from the reaction of CaO (from calcium aluminate cement) with SiO_2_ and Al_2_O_3_ within the transient glassy phase, leading to localized crystallization during the cooling stage.

At 1400 °C, the reference and 5FSC castables showed similar mineralogical patterns. Both were dominated by corundum and mullite, which together accounted for more than 80 wt.% of the total crystalline content. Mullite increased markedly in both materials (44.30 wt.% in the reference and 37.50 wt.% in 5FSC), reflecting extensive formation of secondary mullite. This process is typical of alumina–silica systems at high temperature and promotes the growth of interlocking needle-shaped crystals that strengthen the microstructure [44,56,57,58]. In the 10FSC and 15FSC formulations, mullite reached even higher values (44.59 wt.% and 46.91 wt.%). These compositions also exhibited greater amounts of anorthite (13.81 wt.% and 14.92 wt.%) and retained measurable quartz. The persistence of SiO_2_-rich phases indicates that the total silica content exceeded what was required to fully convert the system into mullite and anorthite. This silica excess favored polymorphic transformations of quartz and cristobalite during heating and cooling. Such transformations involve significant volume changes that increase the likelihood of microcracking and porosity formation, which can negatively affect densification and mechanical properties [13,42].

#### Rietveld Refinement Analysis

A Rietveld refinement was carried out on the four formulations (Control, 5FSC, 10FSC, and 15FSC) at 120 °C, 850 °C, 1050 °C, and 1400 °C to complement the qualitative XRD analysis. This method provided quantitative data for each crystalline phase and allowed a clearer connection between the mineral assemblage and the behavior of the castables after thermal exposure. The refinement results (Table 4) show clear changes in phase composition as the SFS content increases.

Mullite tends to rise in the SFS-bearing mixes, with the 10FSC and 15FSC samples reaching the highest values at 1400 °C (44.59 wt.% and 46.91 wt.%). At the same time, the amount of corundum generally decreases when SFS is added, particularly at the highest temperature. Anorthite becomes more prominent in the 10FSC and 15FSC formulations, reaching 13.81 wt.% and 14.92 wt.% at 1400 °C, which indicates a stronger participation of Ca-containing phases in these mixes. Cristobalite and quartz show only moderate changes, although quartz increases noticeably at intermediate temperatures in SFS-containing samples. The rise in mullite content is especially important because this phase provides high refractoriness, low thermal expansion, and good resistance to thermal and mechanical damage. The results suggest that SFS contributes additional reactive silica that promotes mullite formation during firing. This reaction consumes part of the available alumina, which may explain the lower corundum fractions found in the modified mixes. The increase in anorthite in the higher SFS formulations can be linked to the presence of Ca-rich species in the slag, which favor the formation of Ca–Al–Si phases during heating.

Although the trends observed in the refinements reflect the main reaction pathways, the final phase assemblage is also influenced by the granulometry of the components, the reactivity of the raw materials, and the minor oxides introduced by SFS. These factors may contribute to the variations observed at different temperatures and substitution levels.

### 3.4. Mechanical Characteristics of Refractory Castables

**Figure 8:**
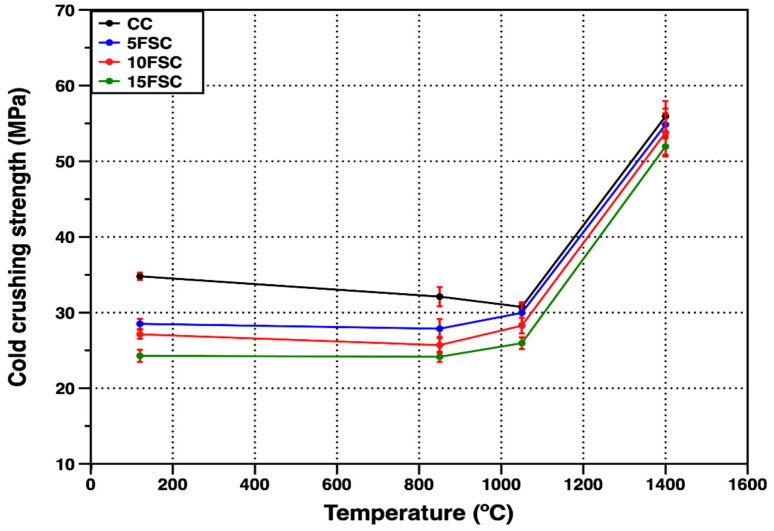
Effect of aluminum spent foundry sand on the cold crushing strength of refractory castables as a function of firing temperature.

Figure 8 presents the cold crushing strength (CCS) values of the reference refractory castable and the SFS-containing formulations as a function of firing temperature. The reference castable exhibited the highest compressive strength at 120 °C (34.8 MPa), primarily due to the hydraulic bonding effect associated with the hydration of Secar 80 cement particles in contact with mixing water [43,59]. During the setting stage, metastable hydration products such as CAH_10_ and C_2_AH_8_ are generated, together with small amounts of amorphous or weakly crystalline AH_3_. These phases provide temporary bonding between particles and give the unfired specimens their initial mechanical strength [60].

In contrast, the SFS-containing castables showed lower initial compressive strengths, with values of 28.5, 27.1, and 24.3 MPa for the 5FSC, 10FSC, and 15FSC compositions, respectively. This reduction can be attributed to the use of a secondary raw material (spent foundry sand) with lower particle density, higher water demand, and weaker particle–matrix interactions during mixing, which together hindered the development of a dense hydraulic network.

As the firing temperature increased to 850 °C, all formulations exhibited a general decline in CCS due to several concurrent transformations. Initially, physically and chemically adsorbed water evaporates, followed by the progressive dehydration of calcium aluminate hydrates. These reactions destabilize the hydraulic bonds, converting CAH_10_ and C_2_AH_8_ into the more stable C_3_AH_6_ phase, which subsequently decomposes at temperatures above 270 °C. The decomposition of amorphous AH_3_ occurs around 100 °C, further weakening the green structure [56,57].

For the SFS-containing castables, an additional volumetric modification arises from the α–β quartz polymorphic transformation occurring near 573 °C, which induces internal stresses due to the abrupt volume expansion of silica particles [45]. The combined effects of hydrate decomposition and quartz transformation promote microcracking and pore development, resulting in lower CCS values of 32.1, 27.8, 25.7, and 24.1 MPa for the CC, 5FSC, 10FSC, and 15FSC castables, respectively.

Upon further heating to 1050 °C, the reference castable exhibited an additional decrease in strength, whereas the SFS-containing samples showed partial recovery. At this stage, the hydraulic bond is completely destroyed, and sintering processes begin to dominate. In the SFS-modified systems, the formation of a SiO_2_-rich liquid phase containing minor oxides (Al_2_O_3_, Fe_2_O_3_, and CaO) promotes diffusion and the early development of a ceramic bond. This liquid phase reacts with the alumina and calcium aluminate phases to form secondary mullite and anorthite crystals, as confirmed by the corresponding diffractograms at 1050 °C [14]. The formation of these crystalline phases from the transient liquid helps fill microstructural voids and close open porosity, improving mechanical strength relative to the reference castable [42].

At the maximum firing temperature of 1400 °C, all castables exhibited a significant increase in mechanical strength. Despite the volumetric transformations associated with silica polymorphism, the SFS-containing formulations reached CCS values comparable to the reference castable, achieving 55.9, 54.8, 53.9, and 51.8 MPa for the CC, 5FSC, 10FSC, and 15FSC samples, respectively [13,61]. This enhanced mechanical behavior is directly related to the formation of interlocking networks of secondary mullite and anorthite crystals that serve as an effective ceramic bond. The secondary mullite crystals, formed in situ from the reaction of alumina with SiO_2_-rich liquid phases, exhibit elongated needle-like morphologies that bridge pores and reinforce the microstructure, contributing to the improved mechanical integrity of the refractory material [44,62].

**Figure 9:**
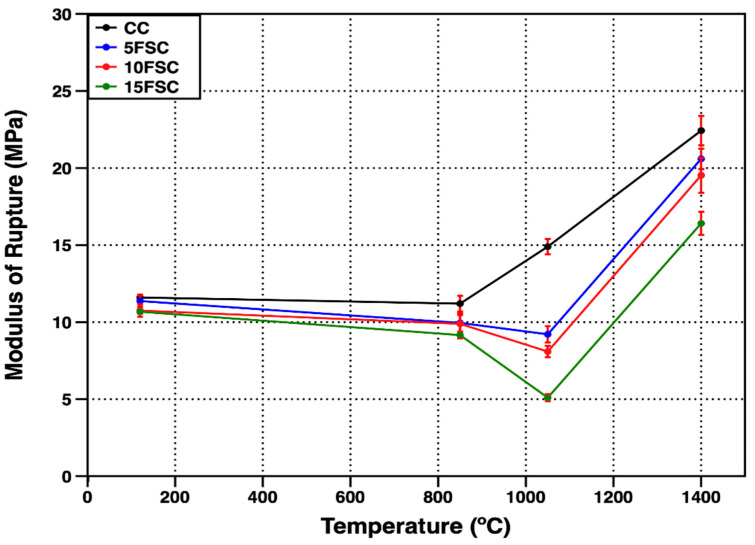
Effect of aluminum spent foundry sand on the cold modulus of rupture of refractory castables as a function of firing temperature.

Figure 9 shows the cold modulus of rupture (CMOR) of the reference refractory castable and the SFS-containing formulations as a function of firing temperature. At 120 °C, the CMOR values were 11.6, 11.4, 10.7, and 10.6 MPa for the CC, 5FSC, 10FSC, and 15FSC castables, respectively. The small decline in flexural strength observed with increasing SFS content at this stage can be ascribed to the higher water demand and reduced particle packing density of the SFS-containing formulations. These factors weaken the hydraulic bonding network formed during setting and drying, which is primarily governed by the formation of metastable calcium aluminate hydrates such as CAH_10_ and C_2_AH_8_ [60].

As the firing temperature increased to 850 °C, a slight reduction in CMOR occurred for all the formulations. This mechanical degradation is attributed to the dehydration and transformation of metastable hydrates, which disrupt the continuity of the hydraulic bonds established during curing. The decomposition of CAH_10_, C_2_AH_8_, and C_3_AH_6_, along with the release of chemically bound water, generates internal microvoids and cracks that compromise flexural resistance.

At 1050 °C, the reference refractory castable exhibited a marked improvement in CMOR, reaching 14.9 MPa. This increase reflects the onset of sintering and the development of a stable ceramic bond between the matrix and the aggregates. However, the SFS-containing refractory castables exhibited lower flexural strengths, with CMOR values of 9.21, 8.09, and 5.10 MPa for the 5FSC, 10FSC, and 15FSC formulations, respectively.

The reduction in mechanical strength is linked to the gradual substitution of flint clay with SFS, which increases the SiO_2_ content of the mixture in both crystalline and amorphous state.

The excess silica undergoes polymorphic transitions during heating, including the reversible α–β quartz transformation at approximately 573 °C and the reconstructive β-quartz–cristobalite transformation at higher temperatures [63]. These transitions involve atomic rearrangements and substantial volume changes, which induce local stresses and microcracking within the refractory matrix. The accumulation of thermally induced microcracks weakens the material’s resistance to flexural stresses, particularly under the combined tensile and compressive loads experienced during CMOR testing. This phenomenon is most evident in the 15FSC formulation, which exhibited the lowest CMOR value (5.1 MPa) due to its higher SiO_2_ content and larger quartz fraction.

At 1400 °C, all formulations showed a significant increase in CMOR, demonstrating that sintering and ceramic bond formation dominated over earlier microcracking effects. The enhanced mechanical response at this stage is attributed to the formation of secondary mullite and anorthite phases, which act as reinforcing agents within the refractory matrix. The interlocked, needle-like secondary mullite crystals, in particular, bridge pores and cracks, improving the material’s load-bearing capacity and overall mechanical integrity [64].

The 5FSC refractory castable achieved a CMOR value of 20.6 MPa, which is comparable to the reference composition (22.4 MPa). This result indicates that a moderate incorporation of aluminum SFS (5 wt.%) can effectively replace part of the flint clay without compromising the mechanical performance of the refractory castable. Such partial substitution offers an environmentally beneficial route to conserve natural aluminosilicate resources while maintaining high-temperature strength and structural reliability.

In contrast, the 10FSC and 15FSC formulations exhibited lower CMOR values of 19.53 and 16.4 MPa, respectively. These decreases are consistent with the XRD results (Figure 6 and Figure 7), which confirmed the persistence of residual quartz and an excess of SiO_2_ after firing at 1400 °C. The volumetric transformations associated with silica polymorphism, coupled with the incomplete sintering of quartz particles, contributed to the microstructural instability and flexural strength reduction observed in these compositions.

### 3.5. Microstructural Characteristics of Refractory Castables

**Figure 10:**
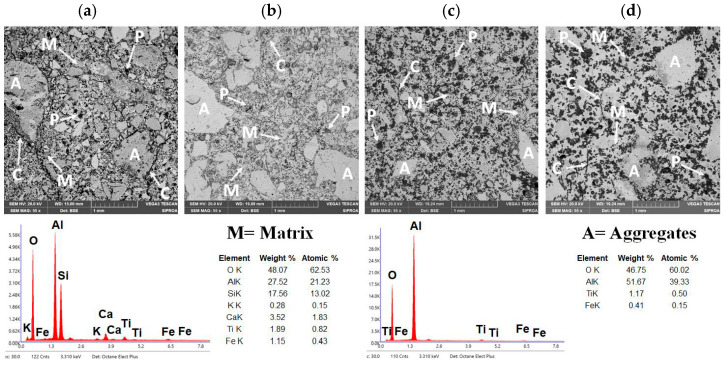
Microstructure of (**a**) the reference refractory castable, (**b**) the 5FSC refractory castable, (**c**) the 10FSC refractory castable, and (**d**) the 15FSC refractory castable developed at 1400 °C. M = Matrix, A = Aggregates, C = Microcracks, and P = Porosity.

Figure 10a–d show the microstructure of the reference refractory castable (CC) and the SFS-containing formulations after heat treatment at the maximum temperature.

Figure 10a shows the microstructure of the reference refractory castable. The physical properties exhibited by the CC refractory castable are consistent with its microstructural features. Specifically, the material shows coarse and medium-sized corundum aggregates “A” with subangular morphology, which begin to interconnect through a refractory matrix “M” composed of fine-grained mullite and anorthite phases [41,65]. However, the microstructure reveals incomplete densification and limited sintering, reflected by weak bonding between aggregates, visible porosity “P,” and microcracks “C” in the refractory matrix. These defects are primarily generated during the dehydration and phase conversion of hydrated compounds upon heating. Nevertheless, the homogeneous distribution of corundum aggregates and the coexistence of primary and secondary mullite, with minor anorthite, as confirmed by EDS elemental analysis, impart satisfactory mechanical performance under compressive and flexural loading, characteristic of conventional aluminosilicate castables [52,53].

Figure 10b presents the microstructure of the refractory castable incorporating 5 wt.% aluminum SFS (5FSC) as well as after the maximum heat treatment. The 5FSC microstructure shows similar features to the reference castable, with coarse and medium-sized corundum aggregates “A” distributed within a dense refractory matrix “M.” EDS analysis confirmed the presence of primary and secondary mullite crystals together with calcium aluminosilicate (anorthite) [44,66,67].

Compared with the CC castable, the 5FSC sample exhibits fewer and narrower microcracks “C” at the aggregate–matrix interfaces. This improvement is attributed to the formation of a SiO_2_-rich liquid phase, which facilitated the development of secondary mullite with an interlocking, needle-like morphology. These elongated crystals, together with anorthite, fill the interparticle voids and enhance the bonding between aggregates. Although a moderate amount of residual porosity “P” with subangular morphology is still present and can act as a stress concentrator during mechanical loading [13], the overall microstructure of the 5FSC castable is denser and more cohesive.

The similarity between the 5FSC and CC microstructures confirms the feasibility of partially replacing up to 5 wt.% of calcined flint clay with aluminum SFS from metallurgical residues to produce high-performance refractory materials. This substitution promotes resource conservation and contributes to sustainable ceramic processing without compromising the mechanical integrity of the final product.

Figure 10c shows the microstructure of the refractory castable containing 10 wt.% aluminum SFS (10FSC). In contrast to the CC and 5FSC castables, significant morphological differences are evident.

The coarse and medium-sized corundum aggregates “A” are no longer observed, which indicates that the excess SiO_2_ detected by EDS promoted the formation of a SiO_2_-rich liquid phase that partially dissolved the corundum particles that normally contribute to mechanical strength [13]. This excessive liquid phase disrupts the particle framework, weakening the load-bearing structure of the material. Additionally, a notable increase in the number of pores “P” and microcracks “C” is observed. These features are linked to the polymorphic transformations of silica (α-quartz → β-quartz → cristobalite), accompanied by abrupt volumetric changes and internal stresses that impair the structural cohesion of the refractory matrix. Consequently, the bonding ability of the mullite–anorthite matrix “M” to hold the remaining corundum particles diminishes, which correlates with the reduced cold crushing strength and cold modulus of rupture previously reported for this composition.

Figure 10d shows the microstructure of the refractory castable containing 15 wt.% aluminum SFS (15FSC). This sample presents a clear contrast to the other formulations. According to the EDS results, the high amount of SiO_2_ leads to the formation of a widespread silica-rich glassy phase. This liquid phase dissolves most of the original corundum aggregates labeled “A,” leaving a largely amorphous matrix with only isolated remnants of the initial aggregates. Large pores labeled “P” and numerous microcracks labeled “C” are also present in the microstructure.

Several factors help explain the presence of these larger pores. SFS contains constituents that release gases or experience structural transitions during firing, and these transformations can enlarge pores in localized areas. The irregular and heterogeneous shape of the SFS particles also reduces packing efficiency in the fresh castable, leaving voids between particles that expand into larger pores after sintering. In addition, partial melting or softening of slag-derived phases during heating can cause movement of the liquid phase and trap gases, which leads to the development of coarse pores in specific regions.

The dominance of the glassy phase in the 15FSC sample reduces the mechanical strength under flexural and compressive loading. This behavior differs from the CC and 5FSC castables, where corundum aggregates are better integrated within a network of crystalline mullite and anorthite, as reported in the literature [13]. The results show that high additions of aluminum SFS promote the formation of silica-rich melts that limit densification and reduce the structural integrity of the refractory castable.

## 4. Discussion

The results obtained for the aluminum SFS-containing refractory castables demonstrate that the progressive substitution of calcined flint clay by aluminum spent foundry sand substantially influences the densification behavior, phase evolution, and mechanical integrity of aluminosilicate systems. Overall, moderate incorporation (≤5 wt.%) of SFS was found to preserve the physical and mechanical performance of the reference formulation, whereas higher additions led to detrimental microstructural instability and strength reduction.

The evolution of phases in the control and SFS-modified refractory castables reflects a sequence of chemical reactions involving alumina, silica, and CaO originating from the calcium aluminate cement. The main thermal transformations and reactions are summarized below.

*Sequence of Main Chemical Reactions in the Matrix*

(i) 120 °C—Formation of metastable hydration products (green body).

During curing, CAC hydration forms CAH_10_, C_2_AH_8_, and AH_3_ gels:

CA + 10H → CAH_10_ (1)

2CA + 11H → C_2_AH_8_ + AH_3_ (2)

These phases act as temporary binders and are confirmed indirectly by the presence of only the raw crystalline phases (mullite, corundum, quartz, cristobalite) in XRD at 120 °C.

(ii) 850 °C—Dehydration of CAC hydrates and initial silica reactions.

The metastable hydrates decompose:

CAH_10_ → CA + Al_2_O_3_ + H_2_O↑ (3)

C_2_AH_8_ → CA + Al_2_O_3_ + H_2_O↑ (4)

Dehydration yields fine Al_2_O_3_ and CaO, which later react with SiO_2_.

Silica begins to soften and partially dissolve into alumina-rich regions.

- Control sample: Reduction in quartz/cristobalite peaks, slight rise in corundum.
- SFS samples: Quartz intensification (due to added SFS silica) and polymorphic quartz → cristobalite transitions begin, especially in 15FSC.

(iii) 1050 °C—Formation of mullite and anorthite.

Silica reacts with alumina and CaO to form mullite and anorthite:

Mullite formation (secondary mullite):

3Al_2_O_3_ + 2SiO_2_ → 3Al_2_O_3_·2SiO_2_ (mullite) (5)

Anorthite crystallization from CaO–Al_2_O_3_–SiO_2_ melt:

CaO + Al_2_O_3_ + 2SiO_2_ → CaAl_2_Si_2_O_8_ (anorthite) (6)

Absence of gehlenite in XRD indicates that the anorthite forms directly from a CaO-bearing liquid phase.

- Control sample: Quartz disappears; cristobalite and anorthite increase.
- 5FSC sample: Clear anorthite peak and stronger corundum due to fine SFS silica enhancing diffusion.
- 10FSC sample: Anorthite intensifies but corundum and cristobalite decrease.
- 15FSC sample: Lower anorthite intensity; excess SiO_2_ forms a viscous glass that limits crystallization.

(iv) 1400 °C—Extensive liquid-phase sintering and mullite growth.

At this stage, mullite forms intensively from silica-rich liquid:

Al_2_O_3_ + SiO_2_ (liquid) → Mullite (needle-like crystals) (7)

- Control and 5FSC samples: Similar phase assemblage dominated by corundum and mullite, with strong mullite reflections due to extensive mullite needle growth.
- 10FSC and 15FSC samples: Excess SiO_2_ results in persistent quartz, strong cristobalite, and higher anorthite content. Silica oversaturation promotes α ↔ β quartz and β ↔ α cristobalite polymorphic transitions that increase microcracking.

*Thermal Transformations per Formulation*

- Control sample:

850 °C: Quartz and cristobalite decrease; corundum increases.

1050 °C: Quartz eliminated; cristobalite and anorthite rise.

1400 °C: Strong mullite formation with densified microstructure.

- 5FSC sample:

850 °C: Slight reduction in cristobalite; quartz persists.

1050 °C: Formation of anorthite from CaO–SiO_2_–Al_2_O_3_ melt; enhanced corundum peaks.

1400 °C: Phase assemblage similar to control but with slightly more cristobalite; well-developed mullite needles support densification.

- 10FSC sample:

850 °C: Quartz reduction and stable cristobalite.

1050 °C: Increase in quartz and anorthite; decreased corundum.

1400 °C: Strong mullite, cristobalite, and anorthite, with persistent quartz due to silica excess.

- 15FSC samples:

850 °C: Strong α → β quartz transformation and cristobalite increase.

1050 °C: Low anorthite crystallization due to formation of a viscous SiO_2_-rich melt.

1400 °C: Quartz, cristobalite, and anorthite coexist with abundant silica-rich glass; extensive polymorphic transitions create microcracking.

*Correlation with XRD and BSE/SEM observations*

- XRD correlation:

Quartz and cristobalite intensity trends match the amount of SFS added and the silica polymorphic transitions (20–23° and 25–27° 2θ). Anorthite peaks at 1050–1400 °C confirm reactions between CaO, Al_2_O_3_, and SFS-derived SiO_2_. Mullite peak sharpening at 1400 °C corresponds to secondary mullite formation during liquid-phase sintering.

- BSE/SEM correlation:

Control and 5FSC sample: Dense microstructures with well-developed mullite needles that bridge pores.

10FSC and 15FSC sample: Presence of silica-rich glassy, partially dissolved corundum grains, and microcracks caused by α ↔ β quartz and cristobalite transitions.

These all observations are consistent with XRD evidence of silica saturation and reduced mullite crystallization.

On the other hand, the gradual reduction in bulk density and corresponding increase in apparent porosity observed with SFS incorporation can be attributed to the combined effects of (i) the lower intrinsic density of SFS compared with flint clay, and (ii) the higher water demand required to ensure workable consistency during casting. These parameters collectively favor the development of residual porosity after drying, as supported by previous studies on the use of industrial by-products in aluminosilicate matrices [7,40,43]. Furthermore, the presence of additional silica from the foundry sand introduces a pronounced sensitivity to the polymorphic transformations of quartz and cristobalite, particularly during the heating–cooling cycles around 573 °C. These transitions are well known to generate volumetric fluctuations that initiate microcracking and hinder the formation of a continuous sintered network [42,45,53].

At intermediate firing temperatures (850–1050 °C), all formulations exhibited a temporary decline in mechanical strength, consistent with the decomposition of metastable calcium aluminate hydrates (CAH_10_, C_2_AH_8_, and C_3_AH_6_) and the associated release of chemically bound water [41,56,57]. The progressive destruction of the hydraulic bonds temporarily weakens the matrix, while the polymorphic transformations of SiO_2_ amplify internal stresses. However, at 1050 °C, the onset of solid-state diffusion and the initial development of ceramic bonds led to a partial recovery of mechanical strength, particularly in the SFS-modified compositions. The appearance of anorthite in the diffractograms of these systems indicates that reactive SiO_2_ from the SFS participated in the formation of calcium aluminosilicate phases, which have been reported to enhance bonding at early sintering stages [14,42,55].

Upon firing at 1400 °C, all refractory castables exhibited a pronounced increase in both cold crushing strength and modulus of rupture, reflecting the establishment of a stable ceramic bond. The microstructural and phase analyses confirmed the formation of secondary mullite and anorthite, which act as reinforcing agents by bridging pores and connecting corundum aggregates through interlocking, acicular crystals [44,56,57,58,64]. This microstructure provides the typical intergranular reinforcement that confers improved thermal and mechanical stability to aluminosilicate refractories [52,53,62]. The 5FSC formulation, in particular, achieved comparable strength to the reference castable, confirming that partial substitution of flint clay by aluminum SFS is technically feasible without compromising performance. This finding aligns with reports that small additions of SiO_2_-bearing residues can promote limited liquid-phase sintering, thereby enhancing densification and structural cohesion [13,50].

However, further increasing the SFS content to 10–15 wt.% produced adverse effects associated with excess silica. The persistence of residual quartz and the enhanced cristobalite formation, as identified by XRD, indicate that the SiO_2_ concentration exceeded the stoichiometric balance required for complete mullite and anorthite crystallization. The resulting silica-rich glassy phase limited solid-state diffusion, inhibited aggregate bonding, and generated volumetric instability during cooling. SEM observations confirmed this behavior, revealing dissolution of corundum aggregates, intergranular cracking, and increased porosity, which are microstructural features commonly associated with the softening and reprecipitation of viscous SiO_2_-rich melts [13,42,67]. Consequently, the flexural and compressive strengths decreased substantially in these compositions, highlighting that excessive SFS incorporation compromises the refractory network’s continuity and mechanical resilience.

Therefore, the reduction in cold strength observed in the SFS-containing castables after firing is closely related to the role of the additional SiO_2_ introduced by the foundry sand. The higher silica content promotes the formation of a viscous SiO_2_-rich liquid phase during heating, which limits solid-state diffusion between Al_2_O_3_ and SiO_2_. As a result, the formation of secondary mullite is restricted. This behavior is consistent with the XRD patterns of the 10FSC and 15FSC formulations, where mullite and anorthite reflections were weaker compared with the reference and 5FSC castables. The reduced crystallization of these phases diminishes the development of a robust ceramic bond during firing.

The excess SiO_2_ also increases the amount of quartz and cristobalite in the matrix. Both phases undergo α ↔ β polymorphic transitions during the thermal cycle, and these transformations generate local stresses that lead to microcracking. BSE micrographs confirmed the presence of microcracks and discontinuities in the SFS-rich compositions, particularly in the 10FSC and 15FSC samples, where the silica content was highest.

Another contributing factor is the partial dissolution of corundum grains. SEM–EDS analysis revealed SiO_2_-enriched regions around corundum aggregates, indicating interaction with the silica-rich melt. This reaction weakens the aggregate–matrix interface, reducing the mechanical contribution of the coarse alumina particles. The higher open porosity measured in these formulations further contributes to the lower cold strength, as the SiO_2_-rich melt may trap gases or produce shrinkage voids upon cooling.

In summary, the decline in cold strength is linked to the combined effects of (i) reduced mullite and anorthite formation, (ii) microcracking caused by silica polymorphism, (iii) weakening of corundum aggregates through partial dissolution, and (iv) increased residual porosity in the SFS-rich castables.

From a materials design perspective, these results reveal a clear compositional threshold between beneficial and detrimental SFS contents. At moderate substitution levels (≤5 wt.%), aluminum SFS contributes reactive silica that promotes secondary mullite formation and controlled liquid-phase sintering. Beyond this limit, the excess SiO_2_ induces polymorphic instability and hinders the consolidation of the ceramic framework. Thus, the balance between silica activity and sintering kinetics emerges as a critical parameter governing the microstructural integrity of these materials.

The environmental implications of these findings are also noteworthy. Incorporating small amounts of aluminum spent foundry sand into refractory castables can reduce reliance on virgin aluminosilicate minerals, contributing to circular economy objectives and lowering the carbon footprint associated with raw material extraction. Moreover, this valorization strategy provides a sustainable route for reusing metallurgical residues that would otherwise require landfill disposal.

Finally, the aluminosilicate refractory castables that contain spent foundry sand (SFS) must be suitable for applications where operating temperatures are in the low to moderate range. These materials are commonly used in biomass-fired boilers, particularly systems that operate below 1100 °C. In this temperature range, castables based on fired clay, mullite, and quartz offer a reliable and economical solution. The quartz present in these formulations promotes the formation of a thin glassy layer during service, and this layer improves resistance to alkali attack, which is a major degradation mechanism in biomass combustion environments. The SFS-modified castables developed in this study maintain the main characteristics required for these applications, including adequate mechanical strength, acceptable porosity, and a stable microstructure formed by mullite and anorthite. The castable with 5 wt.% SFS, in particular, showed physical and mechanical performance comparable to the conventional formulation, which supports its potential use in biomass boiler linings, insulation blocks, burner tiles, and other components exposed to cyclic heating and alkali-rich atmospheres.

Therefore, this work shows that SFS can be introduced into aluminosilicate castables without compromising their essential service properties. The results contribute to the development of sustainable refractory materials that combine waste valorization with the performance demands of industrial systems such as wood-fired and biomass-fueled boilers.

### 4.1. Environmental and Sustainability Implications

The use of aluminum spent foundry sand (SFS) in aluminosilicate refractory castables provides a practical approach for reducing waste from aluminum casting operations and decreasing the environmental impact of refractory production. SFS is generated in large quantities and is often landfilled, which can lead to soil alkalization, dust emissions, and the release of trace elements. Incorporating this material into refractory formulations helps lower disposal demands and supports cleaner waste management practices.

Replacing part of the natural flint clay with SFS also reduces the extraction of non-renewable minerals. The processing of aluminosilicate clays requires significant energy and contributes to CO_2_ emissions, so even a 5 wt.% substitution can moderately decrease the embodied energy and carbon footprint of castable manufacturing. At the same time, the reuse of industrial by-products reduces landfill use and lowers transportation and handling costs.

This valorization pathway supports circular economy goals by linking metallurgical waste streams with refractory production, creating a form of industrial symbiosis. The approach is consistent with Sustainable Development Goals 9 and 12, which emphasize innovation, responsible resource use, and sustainable production systems [68,69].

### 4.2. Novelty Statement

This study provides the first evaluation of aluminum spent foundry sand (SFS) as a partial replacement for calcined flint clay in dense aluminosilicate refractory castables. Although other industrial by-products have been examined in refractory systems, the use of aluminum SFS as an aluminosilicate source has not been previously reported.

The work demonstrates that substituting 5 wt.% SFS produces a castable with physical and mechanical properties comparable to the reference material. This performance is linked to the formation of secondary mullite and anorthite promoted by the SiO_2_-rich liquid phase released from SFS during sintering.

The study also highlights the practical relevance of incorporating a metallurgical waste into refractory formulations, offering a sustainable option that supports waste valorization and reduces the consumption of natural raw materials. In summary, the findings establish aluminum SFS as a viable alternative feedstock for producing environmentally responsible refractory castables.

## 5. Conclusions

Solid by-products produced during ferrous and non-ferrous metal smelting, especially aluminum spent foundry sand (SFS), show considerable potential for use as secondary raw materials in advanced ceramic applications. Despite this, their incorporation into refractory formulations for producing dense castables has received limited attention.

Consistent with sustainable materials engineering practices that seek to conserve non-renewable mineral resources, reduce the disposal of industrial waste, and support circular use of materials, this study examined the partial substitution of fine flint clay with aluminum SFS in a conventional aluminosilicate refractory castable. Four formulations containing 0, 5, 10, and 15 wt.% SFS were prepared by casting and vibration. The main conclusions can be summarized as follows:

Feasible replacement level: The castable containing 5 wt.% SFS (5FSC) exhibited a bulk density of 2.25 g/cm^3^, open porosity of 27.01%, and cold crushing strength of 54.8 MPa. These results are within 2–5% of those of the reference composition (CC: BD = 2.31 g/cm^3^, AP = 25.74%, CCS = 55.9 MPa), confirming that the introduction of a limited amount of aluminum SFS does not significantly impair the physical–mechanical performance. This finding supports its potential as an environmentally sustainable alternative raw material in refractory formulations.

Microstructural development and phase evolution: XRD and microstructural observations revealed that the 5FSC composition developed a composite matrix composed of corundum aggregates embedded in an aluminosilicate phase network of primary and secondary mullite and anorthite. The presence of a SiO_2_-rich glassy phase, derived from the SFS, contributed to improved particle bonding and limited microcrack propagation by promoting partial liquid-phase sintering.

Effect of higher substitution levels: Increasing the SFS content beyond 5 wt.% (10FSC and 15FSC) resulted in a noticeable degradation of densification and mechanical integrity. This behavior is primarily associated with the volumetric instabilities caused by the polymorphic transformations of silica (α-quartz → β-quartz → cristobalite) during thermal exposure, which generate internal stresses and disrupt the ceramic bonding structure.

Overall, the results demonstrate that aluminum spent foundry sand can be effectively valorized as a partial replacement (up to 5 wt.%) for natural flint clay in aluminosilicate refractory castables, offering a promising approach to reduce environmental impact and promote waste circularity in refractory production. Further optimization of particle size distribution, flux composition, and pre-treatment of SFS is recommended to enhance its compatibility and expand its application range in high-performance refractories.

## Figures and Tables

**Table 1:** Design and dosage of refractory mixtures expressed in wt.%.

Refractory Formulation	Flint Clay	Bauxite	Kyanite 48	Secar 80 (CAC)	Microsilica	Spent Foundry Sand	Water
**CC**	40	40	5	12.5	2.5	-	+11
**5FSC**	35	40	5	12.5	2.5	5	+11
**10FSC**	30	40	5	12.5	2.5	10	+11.25
**15FSC**	25	40	5	12.5	2.5	15	+11.5

**Table 2:** Oxide’s composition of primary and secondary starting materials expressed in wt.%.

Oxide	Raw Material					
	Flint Clay	Bauxite	Kyanite 48	Secar 80 CAC	Microsilica	Spent Foundry Sand
**SiO_2_**	47.85	9.18	41.20	0.40	94.33	85.52
**Al_2_O_3_**	43.59	81.17	54.73	78.76	0.78	3.09
**CaO**	0.14	0.12	0.11	17.80	0.72	3.82
**Fe_2_O_3_**	2.23	4.93	1.47	0.61	1.04	3.14
**MgO**	0.22	-	-	0.17	0.61	0.30
**TiO_2_**	2.40	3.29	1.46	-	-	0.07
**K_2_O**	0.65	-	-	-	0.49	0.19
**Na_2_O**	-	-	0.08	1.26	0.19	0.16
**LOI**	2.24	0.21	0.40	0.65	1.46	2.97

**Table 3:** Crystalline phases of the raw materials employed in the design of refractory castables.

	Raw Materials				
**Crystalline Phases**	**Aluminum Spent Foundry Sand**	**Calcined Flint** **Clay**	**Calcined** **Bauxite**	**Kyanite**	**Secar 80**
	Quartz (SiO_2_) ICDD 01-087-2096	Mullite (Al_2.272_Si_0.728_O_4.864_) ICDD 01-083-1881	Corundum (α-Al_2_O_3_) ICDD 01-070-7049	Kyanite (Al_2_SiO_5_) ICDD 01-072-1447	Calcium monoaluminate (CA) ICDD 01-076-7124
		Cristobalite (SiO_2_) ICDD 01-087-2096	Mullite (Al_2.272_Si_0.728_O_4.864_) ICDD 01-083-1881	Quartz (SiO_2_) ICDD 01-087-2096	Calcium dialuminate (CA_2_) ICDD 01-089-3851
		Rutile (TiO_2_) ICDD 01-087-2096	Aluminum titanate (Al_2_TiO_5_) ICDD 01-076-8797	Rutile (TiO_2_) ICDD 01-087-2096	Corundum (α-Al_2_O_3_) ICDD 01-070-7049
			Quartz (SiO_2_) ICDD 01-087-2096		

**Table 4:** Rietveld refinement results of crystalline phases at 120 °C, 850 °C, 1050 °C, and 1400 °C for experimental compositions.

Sample		Corundum	Mullite	Cristobalite	Quartz	Anorthite	sig	rwp
**Control**	**1400 °C**	43.77	44.3	1.2	-	10.7	1.69	10.99
	**1050 °C**	56.45	32.9	2.4	1.11	7.12	1.6	11.3
	**850 °C**	63.94	31.95	1.56	2.53	-	2.02	14.27
	**120 °C**	60.26	30.48	4.42	4.81	-	1.77	12.51
**5FSC**	**1400 °C**	51.27	37.5	1.84	1.46	7.9	2.02	7.47
	**1050 °C**	56.86	26.84	5.2	8.37	2.7	2.83	9.72
	**850 °C**	47.08	34.93	3.51	14.45	-	2.37	8.69
	**120 °C**	57.39	28.37	1.76	12.46	-	2.43	8.39
**10FSC**	**1400 °C**	36.56	44.59	1.97	3.04	13.81	2.33	8.99
	**1050 °C**	45.22	23.35	3.24	20.36	7.8	2.84	11.1
	**850 °C**	58.27	24.42	1.36	15.13	-	3.01	10.44
	**120 °C**	48.49	29.7	0.74	21.06	-	2.59	9.51
**15FSC**	**1400 °C**	35.9	46.91	2.04	0.3	14.92	2.33	8.45
	**1050 °C**	48.73	18.11	6.92	18.44	7.78	2.77	10.12
	**850 °C**	49.33	26.77	1.48	22.41	-	2.63	9.62
	**120 °C**	46.87	32.24	0.94	19.93	-	2.64	9.16

## Data Availability

The original contributions presented in this study are included in the article. Further inquiries can be directed to the corresponding author.
